# Mitochondrial alterations accompanied by oxidative stress conditions in skin fibroblasts of Huntington’s disease patients

**DOI:** 10.1007/s11011-018-0308-1

**Published:** 2018-08-17

**Authors:** Paulina Jędrak, Paweł Mozolewski, Grzegorz Węgrzyn, Mariusz R. Więckowski

**Affiliations:** 1 Department of Molecular Biology, University of Gdańsk, Wita Stwosza 59, 80-308 Gdańsk, Poland; 2 Department of Medical Biology and Genetics, University of Gdańsk, Gdańsk, Poland; 3 Nencki Institute of Experimental Biology, Polish Academy of Sciences, Pasteura 3, 02-093 Warsaw, Poland

**Keywords:** Mitochondria, Oxidative stress, Huntington disease, Fibroblasts

## Abstract

Huntington disease (HD) is an autosomal dominant neurodegenerative disorder manifesting as progressive impairment of motor function and different neuropsychiatric symptoms caused by an expansion of CAG repeats in huntingtin gene (*HTT*). Mitochondrial dysfunction and bioenergetic defects can contribute to the course of the disease, however, the molecular mechanism underlying this process is still largely unknown. In this study, we aimed to determine several mitochondrial parameters in HD fibroblasts and assess their relevance to the disease progression as well as to value mitochondrial pathology in peripheral cells as disease potential biomarker. We showed that HD fibroblasts demonstrate significantly lower growth rate compared to control fibroblasts despite the lack of cell cycle perturbations. In order to investigate mitochondrial contribution to cell growth differences between HD and healthy cells, we provided insight into various mitochondrial parameters. Conducted experiments have revealed a significant reduction of the ATP level in HD fibroblasts accompanied by a decrease in mitochondrial metabolic activity in relation to the cells from healthy donors. Importantly, there were no differences in the mitochondrial membrane potential (mtΔΨ) and OXPHOS complexes’ levels. Slightly increased level of mitochondrial superoxide (mt. O_2_^•-^), but not cytosolic reactive oxygen species (cyt. ROS), has been demonstrated. We have also observed significantly elevated levels of some antioxidant enzymes (SOD2 and GR) which may serve as an indicator of antioxidant defense system in HD patients. Thus, we suggest that mitochondrial alterations in skin fibroblasts of Huntington’s disease patients might be helpful in searching for novel disease biomarkers.

## Introduction

Huntington disease (HD) is an autosomal dominant, neurodegenerative disease caused by a CAG repeat extension in the *HTT* gene, encoding the huntingtin protein (Htt), which results in appearance of a long polyglutamine tract in the gene product. Abnormal expansion of CAG repeats (>40) in the first exon of the *HTT* gene leads to the development of fully symptomatic disease, and the length of the polyglutamine tract in Htt is inversely correlated with age of the disease onset. Huntingtin interacts with more than 200 proteins (Li et al. 2004) as well as with numerous cellular organelles, including the nucleus, endoplasmic reticulum, Golgi complex, synaptic vesicles, and mitochondria (Gutekunst et al. 1998). Depending on subcellular localization of the Htt protein, it has different functions, including regulation of cell survival, trafficking, intracellular transport, endocytosis, signaling processes, and gene transcription (Harjes et al. 2003).

Conformational changes in the mutant huntingtin (mHtt) cause accumulation of the protein in the cytoplasm and the nucleus of neurons. This event is probably responsible for degradation of neurons, however, it is still under debate what is the exact mechanism underlying the cell loss. Selective and progressive wasting of the medium spiny neurons in the striatum and cortex of the brain leads to the appearance of spectrum of symptoms, including motor abnormalities (e.g. chorea movements), psychiatric disturbances (e.g. apathy, depression), and cognitive impairments (e.g. memory impairment, dementia). HD occurs in 5–10 per 100,000 individuals in the Caucasian population, with a higher prevalence in Europe, North America, and Australia, and lower in Asia (Bates and Harper 2002; Pringsheim et al. 2012). No therapy is currently available to reverse the symptoms or delay the onset of HD, and the disease is fatal within 15–20 years after diagnosis (Gusella et al. 1983; The Huntington’s Disease Collaborative Research Group 1993; Roos 2010).

In many neurodegenerative disorders, like Alzheimer’s, Parkinson’s, Huntington’s diseases and amyotrophic lateral sclerosis, mitochondrial impairment and oxidative stress play an important role in the disease progression (Filosto et al. 2011; Chen et al. 2012; Wang et al. 2014). Moreover, data from studies carried out on human HD subjects confirmed that both mitochondrial dysfunction and bioenergetic defects contribute to the course of the disease, although the molecular mechanism by which mHtt affects energy metabolism remains uncertain (Chabi et al. 2003; Quintanilla and Johnson 2009; Reddy et al. 2009). It is tempting to speculate that direct interaction of mHtt with mitochondria results in defective bioenergetics processes (Panov et al. 2002; Orr et al. 2008).

Despite the fact that HD research has been focused on the brain pathology, it is worth to mention that Htt is ubiquitously expressed not only in the central nervous system (CNS) but also in peripheral cells (Sharp et al. 1995; Kegel et al. 2002). HD is associated with numerous mitochondrial alterations in nervous cells, however, peripheral mitochondrial issues are more controversial and there is no clear recognition of the nature of these changes. Years before the onset, glucose uptake and consumption is reduced in the striatum, lactate production in basal ganglia and cortex of patients is elevated, and patients exhibit significant weight loss and muscle wasting (Kuhl et al. 1982; Jenkins et al. 1993; Antonini et al. 1996) (Djoussé et al. 2002; Zielonka et al. 2014). Therefore, one may point out that changes in metabolism are associated with the earliest stages of the disease.

Taking into account the slow progress of the neurodegenerative disorders, it is extremely important to stop the irreversible neuronal loss and to start therapeutic intervention even before the clinical onset. Therefore, there is an urgent need to find new biomarkers for the earliest possible detection of the disease stage. In the present study, we measured several mitochondrial parameters in HD fibroblasts and assessed their relevance to the disease progression in order to verify whether mitochondrial pathology in HD fibroblasts may serve as a potential biomarker, as well as to estimate at which stage of the disease the mitochondrial dysfunction can be observed.

## Material and methods

### Formal and ethical issues

The study was approved by the local Ethics Committee of the Medical University of Gdansk (NKEBN/254/2011 and NKEBN/254–431/2012) and was conducted according to the tenets of the Helsinki Declaration. Written informed consents were obtained from participants prior to the study procedures.

### UHDRS assessment

To study the mitochondrial parameters, biopsies were taken from the skin at the forearm from two groups of subjects: 8 genetically confirmed HD patients with symptoms of the disease, and 7 age- and sex-matched healthy subjects (without a family history of HD). Patients were questioned about their health conditions and subjected to series of tests. Based on the results, patients were assigned to different groups according to Unified Huntington’s Disease Rating. The scale (UHDRS) allows for comprehensive clinical rating of HD severity (Huntington Study Group 1996). The following parameters were assessed in this study: motor function, psychiatric and functional capacity.

The motor section of the UHDRS assesses features of HD with standardized ratings as a time of the motor symptoms duration. Psychiatric features in this study were assessed by the Clinical Global Impressions scale (CGI) which is a well-established rating tool applicable to assess the global severity of illness and change in the clinical condition over time in many psychiatric disorders. Three areas are considered in the CGI: illness severity, global improvement or change, and therapeutic response. The CGI ranges from 1 to 7, and parameters describe clinical symptoms as follows: 1 = normal, not ill at all; 2 = borderline mentally ill; 3 = mildly ill; 4 = moderately ill; 5 = markedly ill; 6 = severely ill; 7 = among the most extremely ill patients (Busner and Targum 2007). The functional assessment was measured by the Total Functional Capacity scale (TFC). The TFC scale comprises occupation of five live items: functional decline, finances, domestic chores, activities of daily living, and level of care. The higher score of the TFC indicates a better functioning in general. Characteristics of the study group from this study is shown in Table 1.

**Table 1:** Characteristics of the healthy individuals (C1 – C7) and HD patients (HD1 – HD8) analyzed in this study. Abbreviations: **M** – male, **F** – female, **CGI** – the Clinical Global Impressions scale, **TFC** - the Total Functional Capacity scale, **NT** – not tested, **NA** – not applicable; detailed information concerning rating scales can be found in the text

Patient type	Sex	Age (years)	Number of CAG repeats	Rating scale		Duration of motor symptoms (years)
				CGI	TFC	
C1	M	43	NT	NA		NA
C2	M	50	NT	NA		NA
C3	W	41	NT	NA		NA
C4	M	51	NT	NA		NA
C5	M	43	NT	NA		NA
C6	W	65	NT	NA		NA
C7	W	42	NT	NA		NA
P1	W	44	43	4	II	6
P2	M	54	43	5	III	13
P3	M	60	40	3	II	4
P4	M	43	42	3	II	3
P5	M	49	42	4	I	2
P6	W	64	42	5	II	5
P7	M	65	39	3	I	6
P8	M	41	43	3	II	17

### Cell cultures and supplements

Fibroblasts were cultured at 37 °C in a humidified atmosphere containing 5% carbon dioxide (CO_2_) in DMEM (Thermo Fisher Scientific Inc., Paisley, UK) supplemented with 10% FBS (Thermo Fisher Scientific Inc., Paisley, UK) and 1% antibiotic/antimycotic solution (Sigma-Aldrich Co. LLC., St. Louis, USA) and cultured on 10-cm plates until they reached confluence. Cells were passaged onto multiwell plates 2 days prior to the measurement of ROS and mitochondrial potential, and the medium was changed the day before the experiment. Cells were harvested by treating with 2 ml of 0.25% Trypsin-EDTA solution (Sigma-Aldrich Co. LLC., St. Louis, USA). All experiments were performed at 5–10 passages.

### The rate of fibroblasts proliferation

To estimate cell growth, the sulforhodamine B (SRB) (Sigma-Aldrich Co. LLC., St. Louis, USA) assay has been performed. SRB binds to basic amino acids present in cellular proteins, and its absorbance is directly related to cell number/mass. Fibroblasts were seeded into 24-well plates (10,000 cells/well) and cultured under standard conditions. The SRB procedure was performed at 2nd and 4th day after seeding. Cells were fixed with 1% acetic acid in 100% methanol (Sigma-Aldrich Co. LLC., St. Louis, USA) at −20 °C overnight. Next, multi-well plates were dried, 250 μl of 0.5% SRB were added to each well and then incubated for 1 h at 37 °C. Wells were washed five times with 1% acetic acid to eliminate SRB not bound to the cells. Multi-well plates were dried again, the protein-bound dye was extracted by addition of 500 μl of 10 mM Tris pH 10 (Sigma-Aldrich Co. LLC., St. Louis, USA) followed by 15 min incubation with gentle mixing in the thermoshaker (BIOSAN, Riga, Latvia). Determination of absorbance was performed in the microplate reader (Tecan Trading AG, Männedorf, Switzerland) at 540 nm. For each cell culture, the slope of cell growth curve was calculated after 2 and 4 days.

### Analysis of cell cycle

Cell cycle propagation was analyzed using the MUSE® Cell Analyzer (Merck Millipore) with a Millipore’s Muse® Cell Cycle Assay Kit (Merck Millipore). Briefly, cells were seeded into 60 mm Petri dishes (3 × 10^5^ cells) in standard DMEM medium supplemented with 10% serum. After overnight incubation, cells were synchronized by using serum starvation for 24 h. Subsequently, cells were released into the cell cycle by addition of 10% serum for 20, 24, 28 or 32 h. After trypsinization, cells were washed with PBS and fixed in 70% ice-cold ethanol. Then, the procedure was followed according to the manufacturer’s instructions. Experiments were repeated in triplicate and the mean of propidium iodide (PI) fluorescence intensity was obtained from 10,000 cells.

### ATP level quantification

To evaluate ATP level in cells cultured with and without glucose in the medium, fibroblasts were seeded into 24-well plates (20,000 cells/well). Next day DMEM medium was replaced with the fresh one containing 5 mM glucose or 5 mM galactose (Thermo Fisher Scientific Inc., Paisley, UK). After 24 h, cells were washed with PBS and the CellTiter-Glo® Luminescent Cell Viability Assay (Promega, Madison, Wisconsin, USA), based on the luciferase reaction, was conducted according to the manufacturer’s protocol.

### Measurement of mitochondrial metabolic activity

Fibroblasts grown in 24-well plates were washed twice with PBS and then incubated in PBS containing 5 mM glucose and 6 μM resosurine (Sigma-Aldrich Co. LLC., St. Louis, USA). This indicator of a fluorogenic oxidation-reduction acts as an intermediate electron acceptor in the electron transport chain without the interference of the normal transfer of electrons. We monitored the intracellular reduction of the dye to the resorufin by the transfer of electrons from NADH to resosurine. The fluorescence was recorded immediately thereafter in a microplate reader (Infinite M200, Tecan, Austria) at 510 nm excitation and 595 nm emission wavelengths. Kinetics of fluorescence insensitivity was measured for 40 min (20 cycles). After measurements cells were fixed overnight for the evaluation of cell number in individual wells using SRB procedure in order to normalize the results by the total cell number.

### Evaluation of mitochondrial membrane potential

Mitochondrial membrane potential (mtΔΨ) was investigated using JC10 probe (Invitrogen, Molecular Probes). Fibroblasts grown in 24-well plates (20,000 cells/well) were washed twice with PBS to remove the medium and then incubated in the presence of 5 μM JC-10 (Invitrogen, Molecular Probes) in PBS containing 5 mM glucose for 45 min in dark at 37 °C. Cells were washed twice with PBS and then, green and red fluorescence values were measured using a multiwell plate reader (Infinite M200, Tecan, Austria) at 485 nm excitation /520 nm emission and at 535 nm excitation/635 nm emission wavelengths respectively.

### Evaluation of cytosolic reactive oxygen species level

The level of cytosolic reactive oxygen species (cyt. ROS) was measured with the use of the ROS-sensitive fluorescent probe CM-H_2_DCF-DA (Invitrogen, Molecular Probes). Fibroblasts grown in 24-well plates were washed twice with PBS to remove the remaining medium and then treated with 2 μM CM-H_2_DCF-DA in PBS containing 5 mM glucose for 30 min. at 37 °C. Next, the cells were washed twice with PBS, and the fluorescence was recorded using a multiwell plate reader (Infinite M200, Tecan, Austria) with excitation and emission wavelengths of 585 nm and 520 nm, respectively. After measurements cells were fixed overnight for the evaluation of cell number in individual wells using SRB procedure in order to normalize the results by the total cell number.

### Evaluation of mitochondrial superoxide level

The level of mitochondrial superoxide (mt. O_2_^•-^) was measured with the use of the ROS-sensitive fluorescent probe MitoSox (Invitrogen, Molecular Probes). Fibroblasts grown in 24-well plates (35,000 cells/well) were washed twice to remove the medium and incubated for 10 min. at 37 °C in the presence of 5 μM MitoSox in PBS containing 5 mM glucose. Next, cells were washed twice with PBS, and the fluorescence was recorded using a multiwell plate reader (Infinite M200, Tecan, Austria) with excitation and emission wavelengths of 510 nm excitation and 595 nm, respectively. After measurements cells were fixed overnight for the evaluation of cell number in individual wells using SRB procedure in order to normalize the results by the total cell number.

### Western-blot analysis

The cell pellets were resuspended in cold lysis buffer (50 mM Tris, pH 7.5, 150 mM NaCl, 1% Triton, 0.1% SDS, 1% sodium deoxycholate) containing a protease inhibitor cocktail (Sigma-Aldrich) and a phosphatase inhibitor cocktail (Sigma-Aldrich), added prior to use. The samples were incubated on ice for 30 min. and then centrifuged at 10,000 x g for 20 min. at 4 °C to remove insoluble cellular material. The protein concentration in the supernatants was determined using the Bradford method. The samples for SDS-PAGE were denatured by reducing Laemmli loading buffer at 95 °C or 45 °C (for detection of OXPHOS subunits) for 5 min. From 10 to 40 μg of protein per sample was separated by SDS-PAGE in 8% or 10% polyacrylamide gels and transferred onto PVDF membranes (BioRad) for 90 min. at 300 mA. The PVDF membranes were blocked using Odyssey Blocking Buffer (Li-Cor, Biosciences) in TBS-T buffer (1: 1) for 1 h. The following primary antibodies were used to detect: individual subunits of mitochondrial respiratory chain complexes, OXPHOS (1: 1500, Total OXPHOS Rodent WB Antibody Cocktail) ab110413; antioxidant enzymes: catalase (1:1000; Abcam ab52477), SOD2 (1: 1000, Abcam ab16956), β-actin (1: 10000, Abcam ab-8227), SOD-1 (1: 2000; Santa Cruz sc-11,407), Anti-GPx-1/2 (1:1000; Santa Cruz sc-30,147), Glutathione reductase (1:1000, Santa Cruz sc-32,886) and mitochondrial content marker, TOMM20 (1: 1000, Santa Cruz sc-11,415) followed by the appropriate secondary fluorescent antibodies labeled with IRdye (1: 5000): IRDye® 800CW Donkey anti-Mouse, IRDye® 600LT Donkey anti-Mouse, IRDye® 670LT Donkey anti-Rabbit, IRDye® 800CW Donkey anti-Rabbit (LI-COR, Inc., Lincoln, USA), in Odyssey Blocking Buffer in TBS-T buffer (1: 1). The relative levels of the detected proteins were visualized using an Odyssey Infrared Imaging System (Li-Cor Biosciences, Lincoln, NE, USA). The density of the bands was analyzed using Image™ Studio software version for the Odyssey® 3021 and Image Studio Lite Ver. 5.2. The quantitative interpretation of Western-blot data in terms of fold changes in protein expression between samples were made by using β-actin as a loading control for each protein. The calculations were based on the differential densitometry ratios of the chemiluminescent signals from the blots of the target protein and the ratio of density of the loading control.

### Statistical analysis

Statistical analyses were performed using Statistica 12 software. Particular statistical test was selected based on the normality of distribution (the Shapiro-Wilk test) and homogeneity of variance (the Levene test). When two independent groups were compared, the *t-*test or U Mann–Whitney test was performed, depending on sample size, distributions, and variance. Principal component analysis (PCA) was performed with the use of “R” software.

## Results

It has been demonstrated previously that HD fibroblasts manifest some of the mitochondrial dysfunction consistent with the neuronal disease phenotype (Sassone et al. 2009; Petersen et al. 2014; Marchina et al. 2014; Jędrak et al. 2017). In the present work, we investigated whether the mitochondrial alterations that could be observed in fibroblasts from patients with HD correlate with the disease severity and may serve as a potential biomarker of HD.

### Fibroblast proliferation

**Fig. 1:**
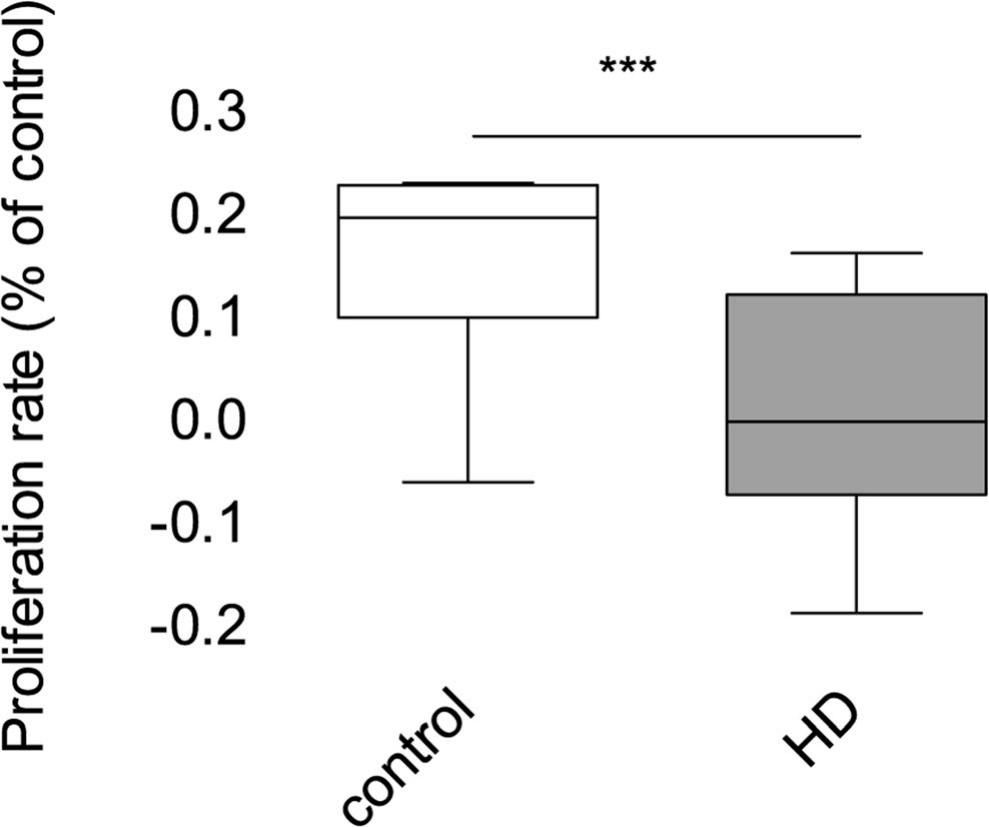
Proliferation of control and HD fibroblasts. The SRB procedure was performed at 2nd and 4th day after seeding. For each cell line, the slope of the proliferation curve was calculated. Box plot represents median, 25 and 75% percentiles, and minimum and maximum. Statistically significant *p*-values were considered when: *p < 0.05, **p < 0.01, ***p < 0.001. The exact p-value is *p* = 0.0176 for HD patients vs. control

To investigate potential differences in cellular physiology between control and patients fibroblasts, we started from analysis of the rate of cell growth. Alterations in the proliferation rate can visualize if HD fibroblasts suffer from metabolic defects. Our results showed that HD fibroblasts were characterized by significant inhibition of growth compared to healthy, control cells (Fig. 1). This growth decline may reflect various cellular alterations, including cell cycle progression and can be a cause of relevant abnormalities in mitochondrial functions, resulting in energetic metabolism impairment.

### Cell cycle

**Fig. 2:**
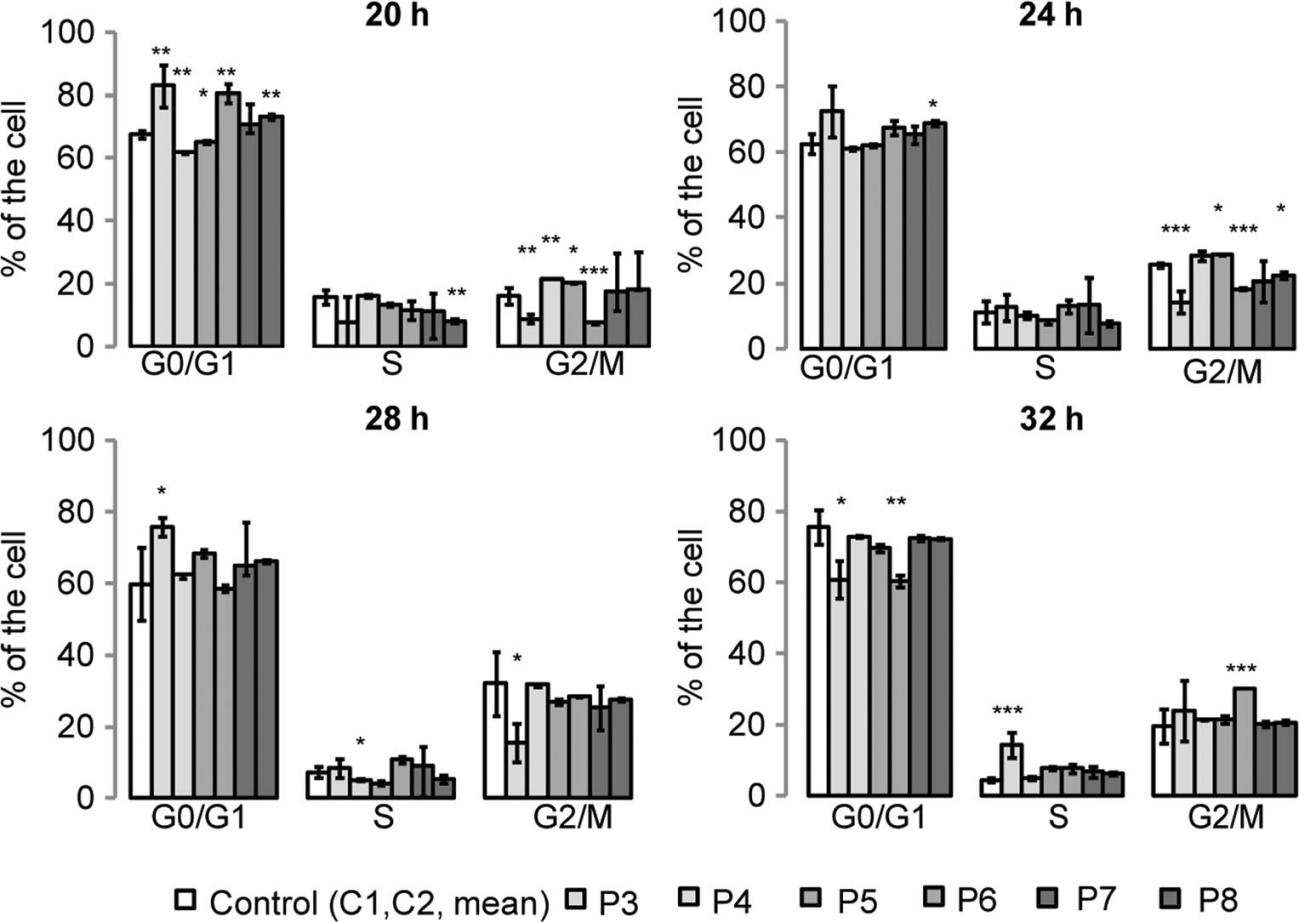
Cell cycle analysis of control and HD fibroblasts. Cells were synchronized by serum starvation for 24 h and released into the cell cycle by addition of 10% serum for 20, 24, 28 or 32 h. Experiments were repeated in triplicate and the mean of propidium iodide (PI) fluorescence intensity was obtained from 10,000 cells. Data shown are means ± SD. Statistically significant *p* values were considered when: *p < 0.05, ** *p* < 0.01, ***p < 0.001. The exact *p* values for each comparison are listed below. 20 h after release the cell cycle; G0/G1: *p* = 0.0071 for P3 vs. control, *p* = 0.0035 for P4 vs. control, *p* = 0.043 for P5 vs. control, *p* = 0.0064 for P6 vs. control, *p* = 0.057 for P7 vs. control, *p* = 0.0068 for P8 vs. control; S: *p* = 0.73 for P3 vs. control, *p* = 0.88 for P4 vs. control, *p* = 0.62 for P5 vs. control, *p* = 0.098 for P6 vs. control, *p* = 0.83 for P7 vs. control; *p* = 0.009 for P8 vs. control; G2/M: p = 0.007 for P3 vs. control, *p* = 0.008 for P4 vs. control, *p* = 0.022 for P5 vs. control, *p* = 0.0083 for P6 vs. control, *p* = 0.68 for P7 vs. control, *p* = 0.23 for P8 vs. control. 24 h after release of the cell cycle: G0/G1: *p* = 0.084 for P3 vs. control, *p* = 0.82 for P4 vs. control, p = 0.79 for P5 vs. control, p = 0.32 for P6 vs. control, *p* = 0.49 for P7 vs. control, p = 0.043 for P8 vs. control; S: *p* = 0.77 for P3 vs. control, *p* = 0.85 for P4 vs. control, *p* = 0.64 for P5 vs. control, p = 0.72 for P6 vs. control, *p* = 0.83 for P7 vs. control, *p* = 0.58 for P8 vs. control; G2/M: *p* = 0.0009 for P3 vs. control, p = 0.68 for P4 vs. control, p = 0.043 for P5 vs. control, *p* = 0.0006 for P6 vs. control, *p* = 0.38 for P7 vs. control; *p* = 0.0044 for P8 vs. control. 28 h after release of the cell cycle: G0/G1: *p* = 0.033 for P3 vs. control, *p* = 0.81 for P4 vs. control, *p* = 0.76 for P5 vs. control, *p* = 0.84 for P6 vs. control, *p* = 0.65 for P7 vs. control, *p* = 0.74 for P8 vs. control; S: p = 0.62 for P3 vs. control, *p* = 0.031 for P4 vs. control, *p* = 0.27 for P5 vs. control, *p* = 0.36 for P6 vs. control, *p* = 0.89 for P7 vs. control, *p* = 0.45 for P8 vs. control; G2/M: *p* = 0.029 for P3 vs. control, p = 0.88 for P4 vs. control, *p* = 0.79 for P5 vs. control, p = 0.81 for P6 vs. control, *p* = 0.69 for P7 vs. control, *p* = 0.73 for P8 vs. control. 32 h after release of the cell cycle: G0/G1: *p* = 0.025 for P3 vs. control, *p* = 0.32 for P4 vs. control, *p* = 0.063 for P5 vs. control, *p* = 0.0089 for P6 vs. control, *p* = 0.78 for P7 vs. control; *p* = 0.72 for P8 vs. control; S: *p* = 0.0007 for P3 vs. control, p = 0.83 for P4 vs. control, p = 0.063 for P5 vs. control, *p* = 0.059 for P6 vs. control, *p* = 0.078 for P7 vs. control, *p* = 0.092 for P8 vs. control; G2/M: *p* = 0.28 for P3 vs. control, *p* = 0.35 for P4 vs. control, p = 0.32 for P5 vs. control, *p* = 0.0008 for P6 vs. control, *p* = 0.47 for P7 vs. control, *p* = 0.52 for P8 vs. control

In order to determine whether the slower proliferation of fibroblasts derived from HD patients could be associated with cell cycle perturbations, cell cycle analysis has been performed. Control and HD fibroblasts were synchronized by serum starvation for 24 h, then, the percentages of cells in G0/G1, S and G2/M phases were assessed after releasing cells into the cell cycle by addition of 10% serum for different periods of time. Most of the observed changes in the percentage of cells in different phases of the cell cycle were present when the cell release occurred after 20 h of starvation (as depicted in Fig. 2). Interestingly, our analysis revealed that, despite the significant inhibition of cell growth and some statistically significant differences in the cell cycle in a single cell line, we could not confirm that HD cells are characterized by a significant impairment of the cell cycle. The lack of relevant changes in the cell cycle propagation in HD fibroblasts suggests the existence of other mechanisms responsible for the slower growth of fibroblasts derived from HD patients.

### ATP level

**Fig. 3:**
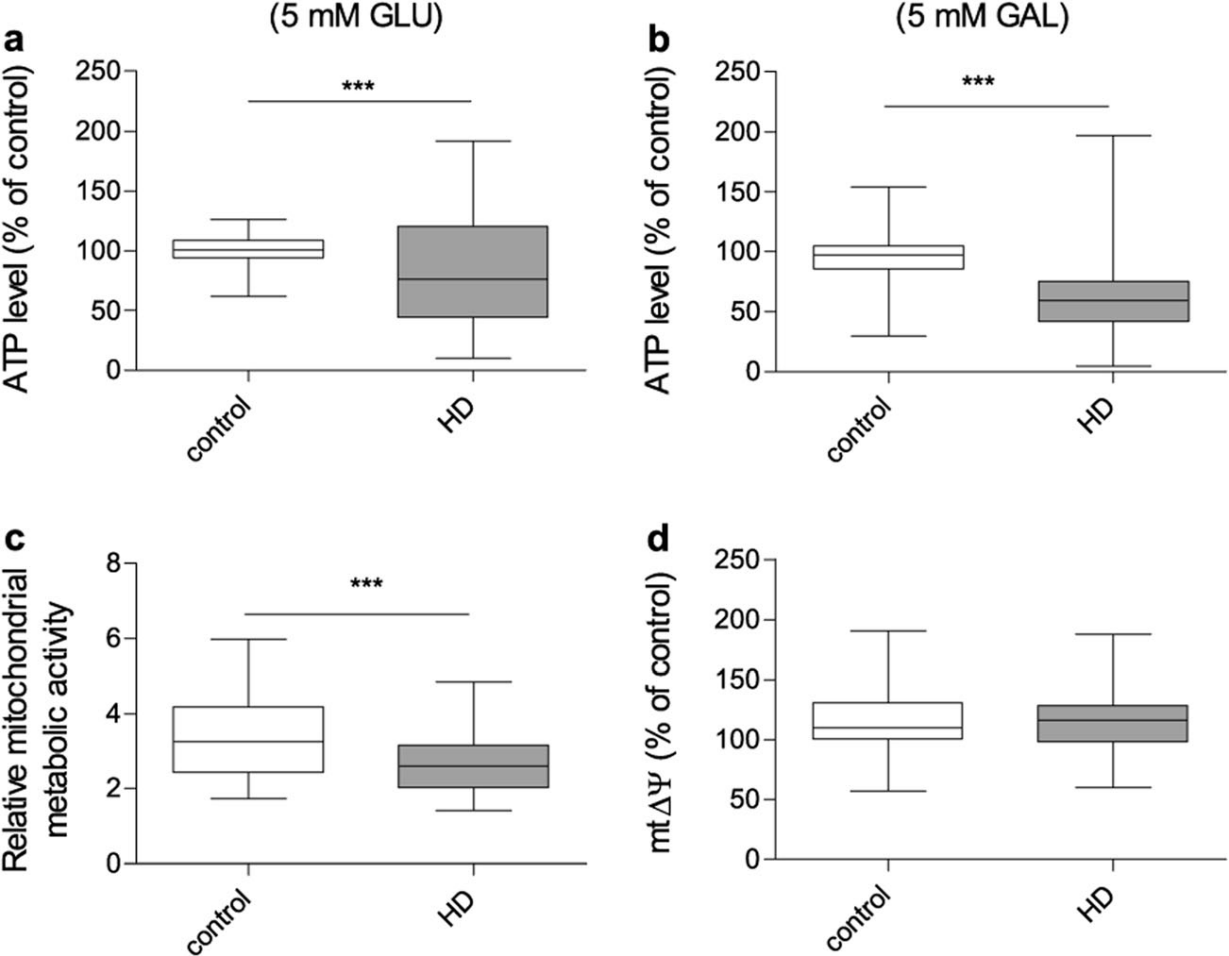
Bioenergetic status of control and HD patient’s fibroblasts. 100% represents the median of analyzed parameters of the control fibroblasts’. Box plot represents median, 25 and 75% percentiles, and minimum and maximum. Statistically significant p values were considered when: *p < 0.05, **p < 0.01, ***p < 0.001. The exact p values for each comparison are listed below. **a** ATP level in the presence of 5 mM glucose in the growth medium (*p* = 0.0006 for HD patients vs. control); **b** ATP level in the presence of 5 mM galactose in the growth medium (*p* = 0.0004 for HD patients vs. control); **c** relative metabolic activity using resosurine test (*p* = 0.0009 for HD patients vs. control) **d** ΔΨ using JC10 probe (*p* = 0.67 for HD patients vs. control)

Since mitochondria are the major source of ATP, produced during oxidative phosphorylation (OXPHOS), changes in the ATP level can suggest mitochondrial defect and can highlight alterations in cellular energy production processes. In order to estimate intracellular ATP level in studied fibroblasts, two different cell culture conditions have been used. Cells were cultured using either DMEM with 5 mM glucose or DMEM without glucose, where galactose served as the main carbon source. In the cells grown in a glucose-free medium, less ATP is produced via glycolysis and energetic metabolism is predominately based on OXPHOS. As presented in Fig. 3a and b, a significant reduction of the ATP level was observed in HD fibroblasts either in the presence of glucose or in the galactose-containing medium. However, under the latter conditions, when cells were cultured in the glucose-depleted medium, the difference in ATP levels between fibroblasts obtained from HD patients and the control was more pronounced. These results may indicate the presence of some alterations in the ATP synthesis machinery and visualize manifestations of mitochondrial dysfunction in HD fibroblasts, which are more pronounced when glycolysis process is restricted.

### Mitochondrial metabolic activity

Slower cell growth, despite the lack of cell cycle perturbations, accompanied with the lower ATP level in HD fibroblasts, could suggest possible alterations in the OXPHOS machinery. To test mitochondrial metabolic activity, the resosurine reduction assay has been used. The cellular compartments where resosurine can be reduced are debated, however, it is believed that this process occurs also in mitochondria, and it is related with the mitochondrial respiratory chain complex I activity. This experimental approach revealed a statistically significant decrease in the resosurine reduction rate in HD fibroblasts in relation to the cells from healthy donors (Fig. 3c). Slower cell growth, significant decrease of cellular and mitochondrial metabolic activity, OXPHOS malfunctioning, and resultant reduced cellular ATP level, may be the reason why HD fibroblasts are more susceptible to damage by toxic factors, like mHtt.

### Mitochondrial membrane potential

The mitochondrial ATP production is strongly dependent on the mitochondrial inner membrane potential (mtΔΨ), thus, we hypothesized that the lower level of ATP observed in HD fibroblasts may be related to the alterations in the mtΔΨ. To address this issue, we evaluated mtΔΨ using JC10 probe in both control and HD cells. Surprisingly, this parameter did not show any differences between fibroblasts derived from HD patients and healthy controls (Fig. 3d).

### The level of individual subunits of the mitochondrial respiratory chain and ATP synthase

**Fig. 4:**
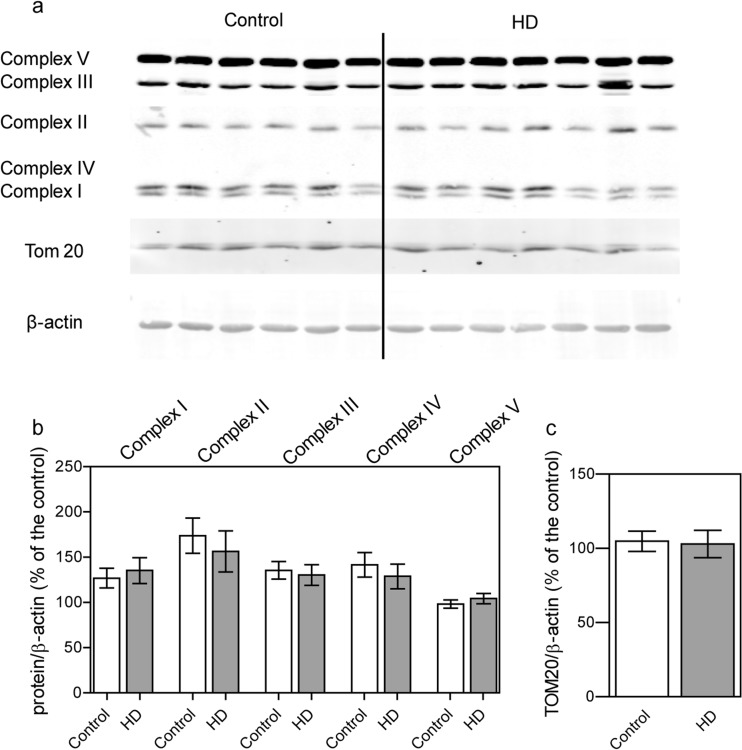
Level of representative subunits of the mitochondrial respiratory chain complexes and TOM20 in control and HD patient’s fibroblasts. Statistically significant *p*-values were considered when: *p < 0.05, **p < 0.01, ***p < 0.001. **a** Representative Western blots of OXPHOS subunits (CV-ATP5A, CIV-MTCO1, CIII-UQCRC2, CII-SDHB, CI- NDUFB). Samples (40 μg protein/lane) were separated on 10% gel. **b** Densitometry analysis of OXPHOS subunits. The level of individual protein was calculated as a ratio to β-actin. Data shown are means ± SD. The exact *p*-values for each comparison are listed below. Complex I, *p* = 0.6526 for HD patients vs. control; Complex II, *p* = 0.3424 for HD patients vs. control; Complex III, *p* = 0.7354 for HD patients vs. control; Complex IV, *p* = 0.5103 for HD patients vs. control; Complex V, *p* = 0.4289 for HD patients vs. control. **c** Densitometry analysis of mitochondrial mass marker TOM20 The level of Tom 20 was calculated as a ratio to β-actin. Data shown are means ± SD The exact p-value is *p* = 0.8754 for HD patients vs. control

Unaltered mtΔΨ does not exclude dysfunction of the OXPHOS machinery in HD fibroblasts. Additionally, results of resazurin assay highlighted that in HD fibroblasts there may be a potential alteration of the mitochondrial respiratory chain activity. Reduced ATP level could be partially explained by lower level of subunits or respiratory chain complexes being a part of OXPHOS. In order to validate this assumption we performed Western-blot analysis of mitochondrial OXPHOS components. This allowed us to check whether decreased levels of respiratory chain subunits might contribute to observed defects in mitochondrial function. We used antibodies against representative subunits of OXPHOS (CV-ATP5A, CIV-MTCO1, CIII-UQCRC2, CII-SDHB, CI- NDUFB8). The conducted analysis did not show any differences in OXPHOS composition between HD fibroblasts and control healthy cells (Fig. 4). Moreover, we evaluated also the level of the TOM20 protein, which can be used as a mitochondrial mass marker. Our results showed that there is no difference in mitochondria content in HD and control fibroblasts.

### The level of reactive oxygen species

**Fig. 5:**
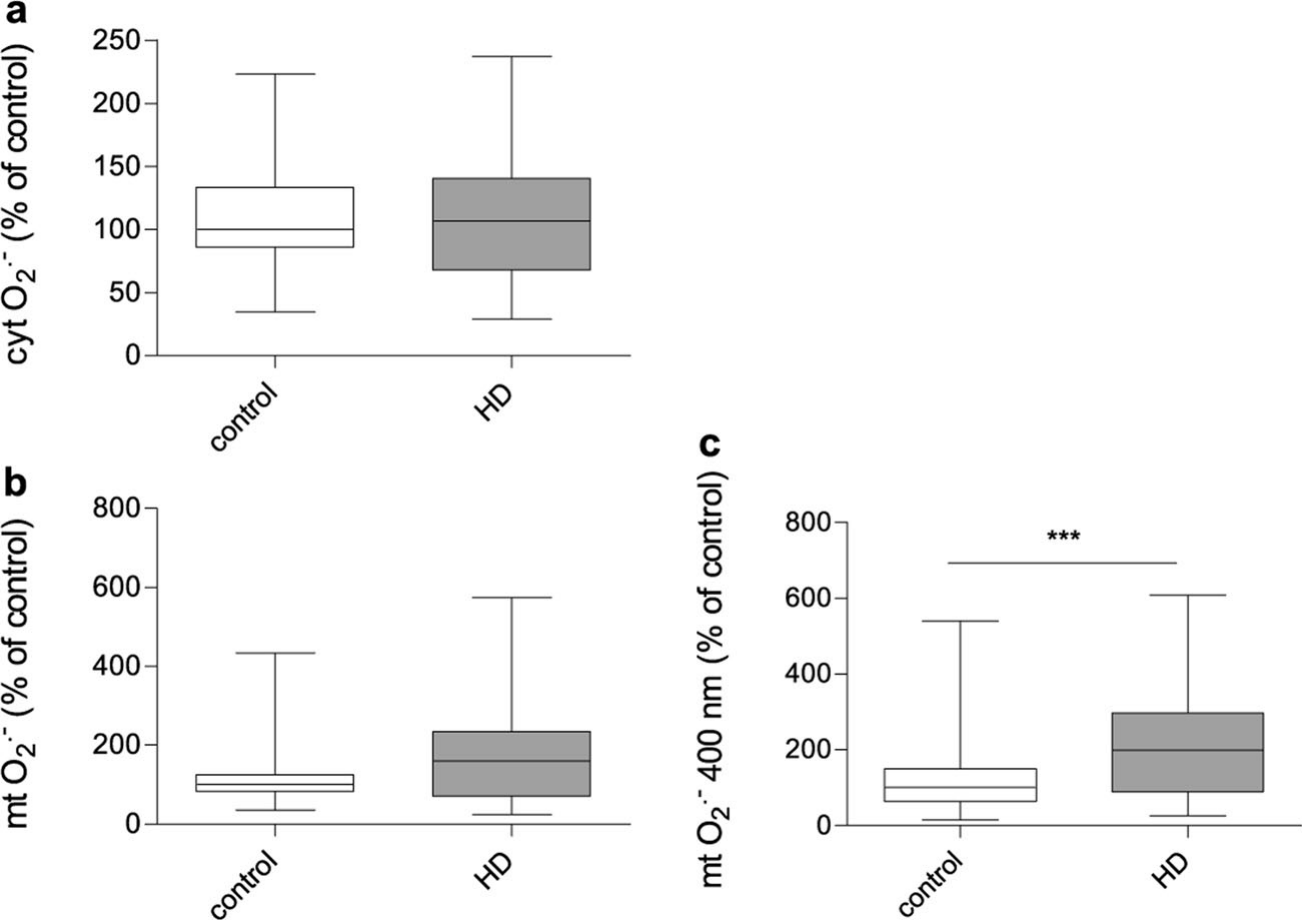
The level of reactive oxygen species in control and HD patient’s fibroblasts. 100% represents the median of analyzed parameters of the control fibroblasts’. Box plot represents median, 25 and 75% percentiles, and minimum and maximum. Statistically significant p-values were considered when: *p < 0.05, **p < 0.01, ***p < 0.001. The exact *p*-values for each comparison are listed below. **a** cytosolic reactive oxygen species level (cyt. ROS) (*p* = 0.92 for HD patients vs. control), **b** mitochondrial superoxide level (mt. O_2_^•-^) (*p* = 0.06 for HD patients vs. control), **c** mitochondrial superoxide level (mt. O_2_^•-^) measured by a specific excitation peak at ~400 nm (*p* = 0.0006 HD patients vs. control)

Even without evident alterations in the OXPHOS composition, we verified whether mitochondrial dysfunction in HD fibroblasts can be accompanied by the increased ROS levels and the status of oxidative stress. We measured the relative amounts of cytosolic ROS and superoxide anion radicals located in the mitochondrial matrix (mt. O_2_^•-^) using CM-H_2_DCF-DA and MitoSOX probes respectively. Interestingly, we found that HD and control fibroblasts had comparable levels of cytosolic ROS (Fig. 5a) as well as mt. O_2_^•-^ (Fig. 5b). What is important for our considerations, MitoSOX probe is oxidised by superoxide to form 2-hydroxymitoethidium, which excites and emits at 510 and 580 nm, respectively (Zielonka et al. 2008; Robinson et al. 2008). Similar to DHE, MitoSOX Red can also undergo unspecific reactions with other oxidants to form mito-ethidium, which overlaps the fluorescence peak of 2-hydroxymitoethidium (Zielonka and Kalyanaraman 2010; Kalyanaraman et al. 2012). Interestingly, it has been shown that the superoxide-specific product of MitoSOX Red oxidation has a specific excitation peak at ~400 nm (Robinson et al. 2008). We found that, when ROS was measured at these special conditions, mt. O_2_^•-^ level was significantly elevated in fibroblasts derived from HD patients comparing to the healthy counterparts (Fig. 5c), what indicates possible existence of oxidative stress in HD fibroblasts.

### The status of the antioxidant defense system

**Fig. 6:**
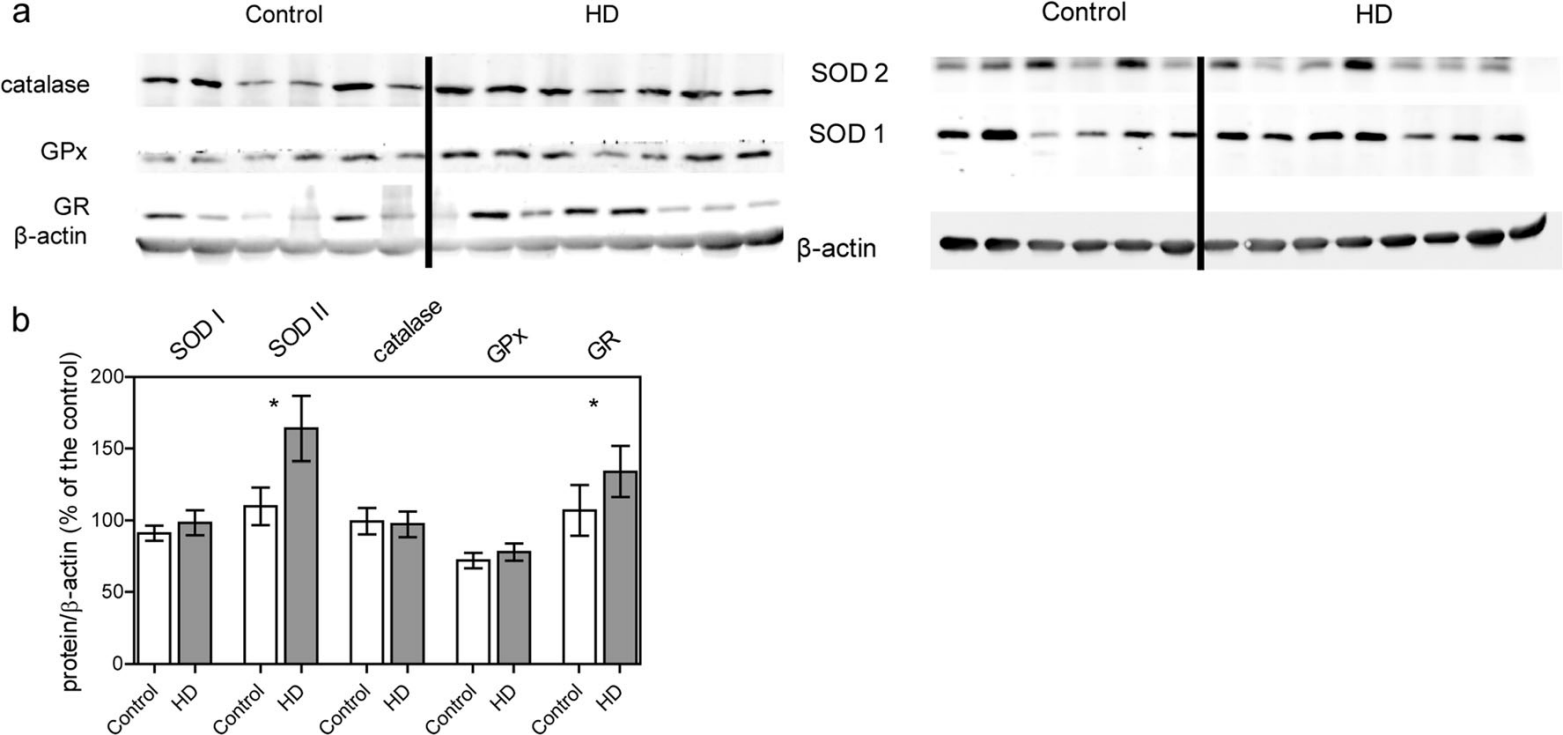
Level of antioxidant enzymes in control and HD patient’s fibroblasts. Statistically significant p-values were considered when: *p < 0.05, **p < 0.01, ***p < 0.001. **a** Representative Western blots of antioxidant enzymes (SOD1,SOD2,catalase, GPx,Gr). Samples (35 μg protein/lane) were separated on 10% gel. **b** Densitometry analysis of antioxidant enzymes level. The level of individual protein was calculated as a ratio to β-actin. Data shown are means ± SD. The exact p-values for each comparison are listed below. SOD1, *p* = 0.76 for HD patients vs. control; SOD2, *p *= 0.04 for HD patients vs. control; catalase, *p* = 0.65 for HD patients vs. control; GPx, *p* = 0.48; Gr, *p* = 0.032 for HD patients vs. control

The ROS level, measured with the use of fluorescent probes, depends on the ratio between the rate of ROS production and the efficiency of antioxidant defense system. Therefore, levels of free radicals in the cell are closely related to the levels and activities of antioxidant enzymes. To examine whether observed increased mt. O_2_^•-^ level is associated with higher production or with insufficient antioxidant defense machinery, we measured levels of antioxidant enzymes, including superoxide dismutases (SOD1 and SOD2), glutathione peroxidase (Gpx), glutathione reductase (GR) and catalase (CAT) (Fig. 6). We found a significantly increased SOD2 level (located within the mitochondrial matrix) in HD fibroblasts. Furthermore, we noticed an elevated level of GR (located mainly in the cytoplasm) which catalyzes the reduction of glutathione disulfide (GSSG) to the sulfhydryl form (GHS). These results indicate that the upregulation of individual elements of antioxidant defense system in HD patients could be a cellular response to the elevated mt. O_2_^•-^ level to counteract its negative prooxidant effect on mitochondria.

### Principal component analysis (PCA) showing differences in the measured parameters in term of scales describing the motor, mental and cognitive disease severity

HD is a multi-symptomatic disease and progresses at different rates and with different intensity of physical, psychological and cognitive symptoms. Analysis concerning the progress of the disease should be based on a scale describing all types of symptoms. Only such an approach may be appropriate in the search for a universal biomarker of this disease. In our study, we have chosen the scales that reflect the condition of the patient most precisely and with the highest certainty: duration of the motor symptoms, CGI, TFC. In order to verify whether some changes between slightly, moderately and clearly sick fibroblasts donors are present, we decided to classify studied cell lines according to the CGI parameters described for HD patients. It is important to mention, that among the analyzed cell lines, differences were observed (however, not statistically significant) between the subjects classified to the same group in terms of CGI score.

**Fig. 7:**
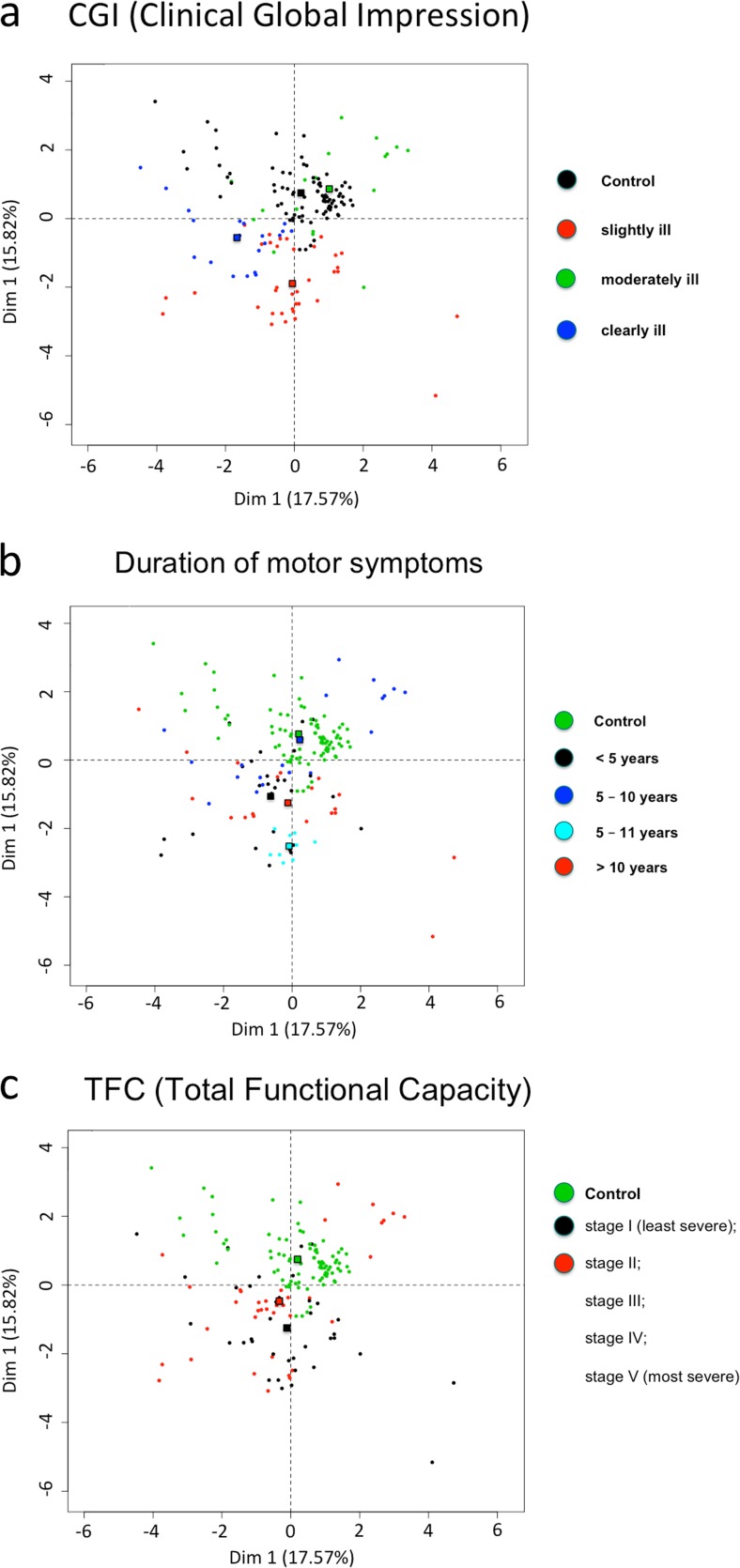
Principal-component analysis showing that fibroblasts from healthy donors and HD patients‘fibroblasts have characteristic and different profiles of investigated parameters when taken into consideration (**a**) duration of the motor symptoms; **b** CGI and **c** TFC. 2D graph of variables PC1 and PC2 created based on 13 parameters measured in fibroblasts derived from HD patients and controls (mtΔΨ; mitochondrial metabolic activity measured with resazurin; the level of ATP estimated in the presence of glucose; the level of intracellular ATP estimated in the presence of galactose; the level of mitochondrial ROS measured with MitoSOX at excitation peak at ~510 nm; the level of mitochondrial superoxide (mt. O_2_^•-^) measured with MitoSOX at excitation peak at ~400 nm; the level of cytosolic ROS measured with CM-H_2_DCF-DA; the level of CAT; the level of SOD1; the level of SOD2; the level of GPx1/2; the level of GR, and the number of mtDNA copies)

To elucidate if based on the parameters investigated in this work,we can show differences between HD and control fibroblasts, a principal component analysis (PCA) was performed using the “R” software. Obtained data were correlated with various scales describing the motor, mental and cognitive disease severity. We used three UHDRS scales as follows: duration of the motor symptoms, CGI, and TFC. We took into consideration 13 parameters measured in fibroblasts derived from patients and controls (mtΔΨ; mitochondrial metabolic activity measured with resosurine; the level of ATP estimated in the presence of glucose; the level of intracellular ATP estimated in the presence of galactose; the level of mitochondrial ROS measured with MitoSOX at excitation peak at ~510 nm; the level of mitochondrial superoxide (mt. O_2_^•-^) measured with MitoSOX at excitation peak at ~400 nm; the level of cytosolic ROS measured with CM-H_2_DCF-DA; the level of CAT; the level of SOD1; the level of SOD2; the level of GPx1/2; the level of GR, and the number of mtDNA copies measured in our previous studies). The PCA enabled us to perform a linear transformation of the 13 variables into a lower dimensional space, which retain maximal amount of information about the individual variables (parameters). Interestingly, PCA showed noticeable differences between created profiles characteristic for duration of the motor symptoms, CGI and TFC scales (Fig. 7a, b and c).

## Discussion

There is some strong evidence that mitochondrial abnormalities may have a direct or indirect impact on the pathogenesis of many central nervous system diseases including HD. Despite the fact that HD is a neurodegenerative disease, many alterations are observed throughout the body. Therefore, studies on energy metabolism concerning peripheral cells may have high potential to find new therapeutic targets as well as to develop new biomarkers of the disease. On the other hand, changes in mitochondrial parameters in peripheral tissues are still insufficiently understood. Some of the metabolism-related alterations, like weight loss despite unchanged caloric intake, have been shown in HD patients before the clinical onset of the disease, suggesting that metabolic alterations may be a part of an early cascade of events promoting HD pathogenesis (Mochel et al. 2007). Taking this into account, detailed characteristics of changes in the cell metabolism and mitochondrial metabolic activity in peripheral tissues, which may serve as a potential material in the search for a biomarker, seems to be even more important.

The first evidence highlighting differences between HD fibroblasts and fibroblasts from healthy individuals came from the observation that HD cells have grown significantly slower than control cells. There may be many reasons for the slower growth of these cells, like changes in cell cycle or metabolic disturbances. To check whether HD cells have a disturbed cell cycle, we conducted analysis on synchronized cells cultures. Our results show an obvious slowdown in cell growth and after exclusion of cell cycle abnormalities, it is clear that other mechanisms must be responsible for this effect (Figs. 1 and 2).

Cellular metabolism has been proposed as one of the regulators of cell proliferation, growth, and survival. Many previous in vitro studies suggested a decreased ATP production (Gines et al. 2003; Seong et al. 2005; Wang et al. 2009) and a loss of mitochondrial membrane potential (Snyder et al. 1999; Panov et al. 2002) in models expressing mHTT. In our study, the level of ATP, which is a sensitive indicator of possible bioenergetic disturbances, turned out to be reduced regardless of used culture medium containing different source of carbon. ATP synthesis is tightly regulated by the mtΔΨ which in healthy cells is maintained by the respiratory chain activity. Thus, ATP depletion might be a result of decreased mtΔΨ in HD fibroblasts, however, in our study this parameter was not significantly different when measured in HD and control fibroblasts (Fig. 3). These phenomena may indicate the presence of some compensatory mechanism which allow to maintain proper polarization of the mitochondrial membrane despite defects leading to decreased ATP level.

Symptomatic individuals have decreased levels of complexes II, III and IV in different brain regions (Parker et al. 1990; Gu et al. 1996; Browne et al. 1997; Arenas et al. 1998). In the present work, we showed a decreased mitochondrial metabolic activity in HD fibroblasts, which may indicate the presence of some respiratory chain defects. Western-blot analysis of the levels of representative subunits of OXPHOS complexes did not show any significant differences between studied cells. Our results confirmed previously published data that there are no significant differences in activities of respiratory chain complexes in HD fibroblasts compared to the controls and provided a solid proof for a lack of OXPHOS defects in HD skin fibroblasts (del Hoyo et al. 2006). Normal respiratory chain function was also observed in a presymptomatic HD mice (Guidetti et al. 2001), and together with our results, it could suggest that defective respiratory chain is a secondary feature in the pathogenesis of the disease (Browne 2008). Moreover, to exclude that alterations in the mitochondrial function could be a result of lower mitochondrial content in HD fibroblasts, we analyzed the level of the TOM20 protein. Western-blot analysis showed that mitochondrial content in HD fibroblasts does not differ from the value estimated in fibroblasts from healthy donors. This confirms that decreased mtDNA level, showed in our previous study (Jędrak et al. 2017), is not related to the changes in total mitochondria mass/content (Fig. 4).

Increased oxidative stress very often accompanies metabolic alterations and has an impact on HD progression. Increased ROS production in HD was previously well established in many different disease models which highlighted its role in the pathogenesis (Firdaus et al. 2006; Hands et al. 2011; Chiang et al. 2012; Valencia et al. 2013). In the present study, we showed a significant increase in mitochondrial, but not cellular, ROS levels (Fig. 5). Moreover, we showed elevated levels of two antioxidative enzymes: SOD2 and GR in HD fibroblasts, probably as a cellular response to the increased mt. O_2_^•-^ level (Fig. 6). This fact can be an indication of the presence of the oxidative stress in the HD dermal fibroblasts. Another study conducted with the use of HD fibroblasts revealed a decreased catalase activity (the activity of other antioxidant enzymes remained unchanged) (del Hoyo et al. 2006). It is necessary to mention that the level of individual antioxidant enzymes does not inform about the protein activity which depends on protein modifications, regulation and various other conditions. In the future studies this aspect should be more deeply investigated. Interestingly, the results of other studies (investigating antioxidant strategies) were also inconclusive and depended on analyzed material (Johri and Beal 2012; Ribeiro et al. 2012; Mason et al. 2013). Importantly, in our study only a slightly increased level of mt. O_2_^•-^, measured in mitochondrial matrix may be a result of significantly elevated SOD2 level as a protective mechanism against free radical-induced damage.

Based on our PCA analysis, we can conclude that metabolic dysfunctions in the HD fibroblasts are more visible in early carriers. Usually, psychological complaints are the first symptoms that occur in the disease, and in our study the scale concerning the mental problem (CGI) most strongly correlates with the investigated mitochondrial parameters. Using CGI scale the most prominent changes of mitochondria parameters are observed in mildly mentally ill patients. It is extremely relevant as some results suggest the presence of mitochondrial dysfunction observed in mental disorder like schizophrenia (alterations in brain energy metabolism, electron transport chain activity, and expression of genes involved in mitochondrial function) and may emphasize its physiological value (Prabakaran et al. 2004; Manatt and Chandra 2011; Rajasekaran et al. 2015). To study such correlations it is important to take into consideration that the TFC is a self-reporting test and as previously indicated may not be suitable for patients at the earliest stage of the disease (Tabrizi et al. 2009; Beglinger et al. 2010). Moreover, physical manifestations most often appear as the last symptoms but are measured with the greatest accuracy and certainty. It should be also emphasized that they do not represent the overall duration of the disease.

Interestingly, there are contradictory results of studies on mitochondrial complexes of HD patients, published in the literature. Parker et al. (1990) measured activities of such complexes, and found that complex I, but not other complexes, is less active in platelet HD mitochondria, relative to healthy controls. On the other hand, Gu et al. (1996) found defects in complexes II, III, and IV in HD caudate nucleus, however, no changes relative to controls were detected in platelet mitochondria of HD patients. Contrary to those findings, in another study, decreased activities of complex I were measured in muscles of HD patients (Arenas et al. 1998). Another discrepancy appeared when platelet mitochondria were studied again, and normal activity of complex I was determined in HD patients (Powers et al. 2007). No changes in complexes I to IV were also reported in muscles of persons suffering from HD (Turner et al. 2007). It is difficult to judge wat is the reason for discrepancy of the results mentioned above. One might speculate that the factors affecting activities of mitochondrial complexes in HD patients are as follows: (i) kind of investigated tissue, (ii) severity of disease, (iii) the number of CAG repeats in the *HTT* gene, and (iv) stage of the disease at which samples were withdrawn. At least some of these factors can also affect results of measurements reported in this paper.

In conclusion, we suggest that mitochondrial alterations in skin fibroblasts of Huntington’s disease patients might be helpful in searching for novel disease biomarkers. Particularly, such markers could be useful in preliminary assessments of effects of potential drugs for HD. This might be of special interest in the light of recent proposals of development of novel therapies for HD (Pierzynowska et al. 2018a) and suggestions of potential drugs (Pierzynowska et al. 2018b).

## Conclusions

In summary, the present study describes bioenergetic abnormalities, increased mitochondrial ROS level and upregulated antioxidant defense system in HD fibroblasts. Importantly, our results are in line with the previous study indicating the presence of an early hypermetabolic state in HD patients. However, how exactly mHtt interferes with energy status of the cells remains unknown. In our study, we used skin fibroblasts which seem to be the proper model in the case of screening for mitochondrial disease biomarkers. This work presents approaches and methods that can be used in field of searching early disease biomarkers in HD patients.
